# Biosynthesis and metabolic engineering of isoflavonoids in model plants and crops: a review

**DOI:** 10.3389/fpls.2024.1384091

**Published:** 2024-06-25

**Authors:** Lijun Wang, Chaofeng Li, Keming Luo

**Affiliations:** ^1^ School of Life Science and Engineering, Southwest University of Science and Technology, Mianyang, China; ^2^ Maize Research Institute, Southwest University, Chongqing, China; ^3^ Engineering Research Center of South Upland Agriculture, Ministry of Education, Chongqing, China; ^4^ Chongqing Key Laboratory of Plant Resource Conservation and Germplasm Innovation, Integrative Science Center of Germplasm Creation in Western China (Chongqing) Science City, School of Life Sciences, Southwest University, Chongqing, China; ^5^ Key Laboratory of Eco-environments of Three Gorges Reservoir Region, Ministry of Education, School of Life Sciences, Southwest University, Chongqing, China

**Keywords:** isoflavonoids, biosynthesis, metabolic engineering, bio-function, regulation

## Abstract

Isoflavonoids, the major secondary metabolites within the flavonoid biosynthetic pathway, play important roles in plant defense and exhibit free radical scavenging properties in mammals. Recent advancements in understanding the synthesis, transport, and regulation of isoflavonoids have identified their biosynthetic pathways as promising targets for metabolic engineering, offering potential benefits such as enhanced plant resistance, improved biomass, and restoration of soil fertility. This review provides an overview of recent breakthroughs in isoflavonoid biosynthesis, encompassing key enzymes in the biosynthetic pathway, transporters influencing their subcellular localization, molecular mechanisms regulating the metabolic pathway (including transcriptional and post-transcriptional regulation, as well as epigenetic modifications). Metabolic engineering strategies aimed at boosting isoflavonoid content in both leguminous and non-leguminous plants. Additionally, we discuss emerging technologies and resources for precise isoflavonoid regulation. This comprehensive review primarily focuses on model plants and crops, offering insights for more effective and sustainable metabolic engineering approaches to enhance nutritional quality and stress tolerance.

## Introduction

Isoflavonoids, a major subclass of flavonoids, play pivotal roles in plant growth, development, and stress defense, with recognized implications for human health (Dixon, 1999; Veitch, 2013; Wang et al., 2022). In plant-microbe interactions, isoflavonoids function as signal molecules perceived by microorganisms (Biala-Leonhard et al., 2021). They act as phytoalexins, inhibiting the growth and reproduction of bacteria and fungi, fortifying plant defense against pathogens. Simultaneously, isoflavonoids attract rhizobia to legume root nodules, establishing symbiotic relationships that enhance plant growth, reduce nitrogen fertilizer use, and improve soil fertility (Abd-Alla et al., 2023). Furthermore, the synthesis and accumulation of isoflavonoids in plants are induced by various biotic and abiotic stresses, enhancing overall adaptability (Dixon and Paiva, 1995). Due to their structural and functional resemblance to endogenous estrogen, isoflavonoids and their derivatives are recognized as phytoestrogens, finding applications in functional foods, nutraceuticals, and medicine for disease prevention and treatment (Dixon, 2004; Griffiths et al., 2014; Yu et al., 2021).

With a profound understanding of the significance of isoflavonoid compounds, current research has increasingly emphasized the biosynthesis, transport, and regulation processes in plants. This emphasis has significantly propelled advancements in metabolic engineering and *de novo* synthesis studies. While isoflavonoids are predominantly found in leguminous plants, their scarcity in other plants has led researchers to explore metabolic engineering and synthetic biology as promising strategies. These approaches address the challenges posed by the low abundance and difficulty in obtaining large quantities through conventional crop-based manufacturing or chemical synthesis. Prior reviews have provided detailed reviews of the synthesis, regulation, and metabolic engineering of isoflavones (Yu and McGonigle, 2005; Sohn et al., 2021). However, as time progresses, the functions of an increasing number of genes are being reported, and the application of newer technologies are spurring additional breakthroughs in the study of isoflavonoid synthesis and metabolic engineering. This review will focus on a wider variety of isoflavonoid compounds.

## The chemical structures and functions of various isoflavonoids

**Figure 1:**
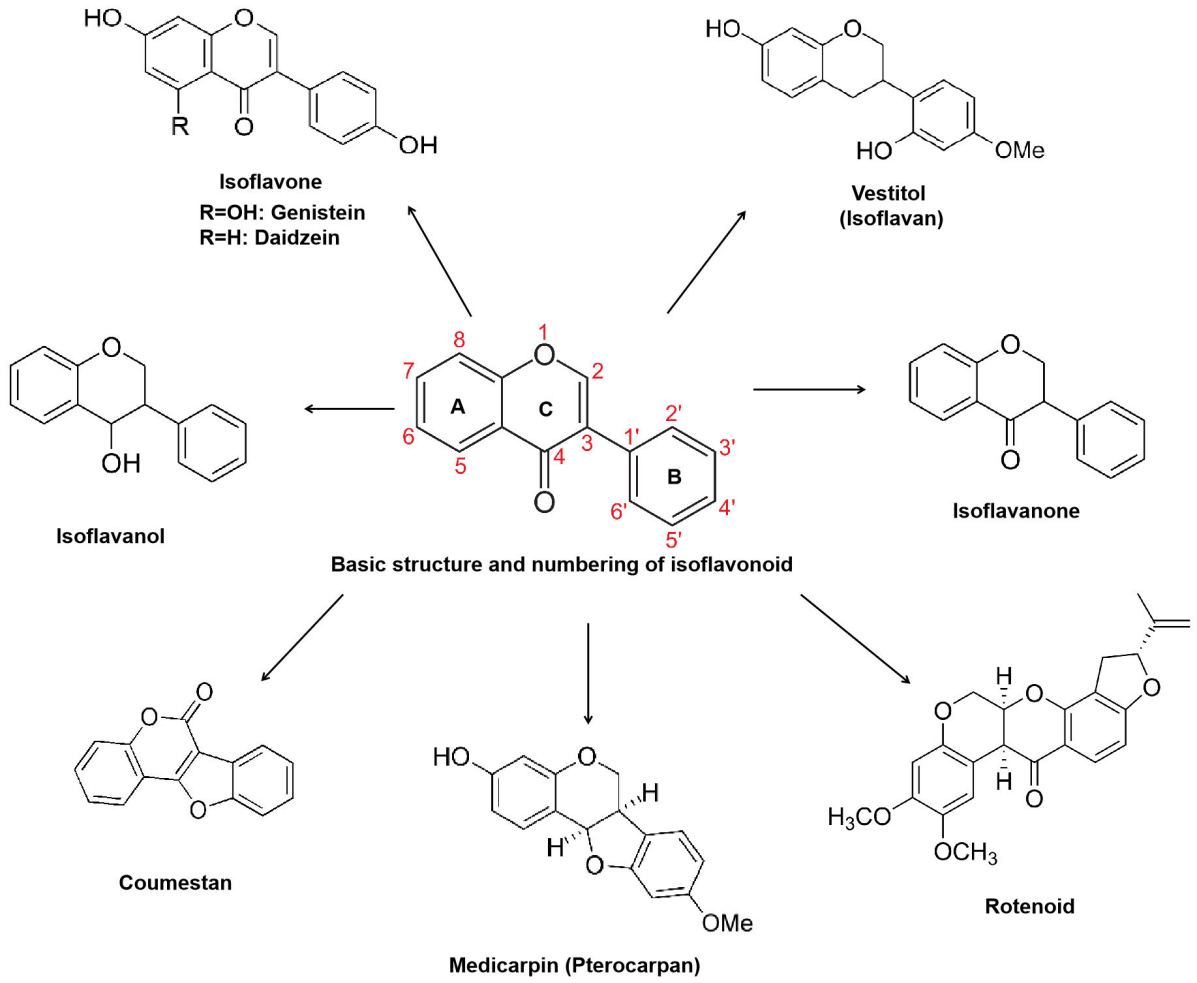
Structures and classification of isoflavonoids. This basic C6-C3-C6 structure consists of a benzene ring (A-ring), a pyran ring (C-ring), and a phenyl group (B-ring).

Isoflavonoids, along with flavonoids, lignins, coumarins, and stilbenes, all belong to the class of phenylpropanoid compounds, which are crucial for plant adaptation to terrestrial environments, enabling plants to withstand gravity and offering protection against UV radiation, desiccation, pathogens, and herbivores (Muro-Villanueva et al., 2019; Yadav et al., 2020; Dong and Lin, 2021). Isoflavonoids, also known as 3-phenylchromanes, feature a C_6_-C_3_-C_6_ backbone with the phenyl B-ring attached to position 3 of the heterocyclic pyran ring (the C ring). Based on structural characteristics, they are classified into isoflavones, isoflavans, pterocarpans, rotenoids, coumestans, and derivatives formed through methylation, glycosylation, and acylation (**Figure 1**).

**Figure 2:**
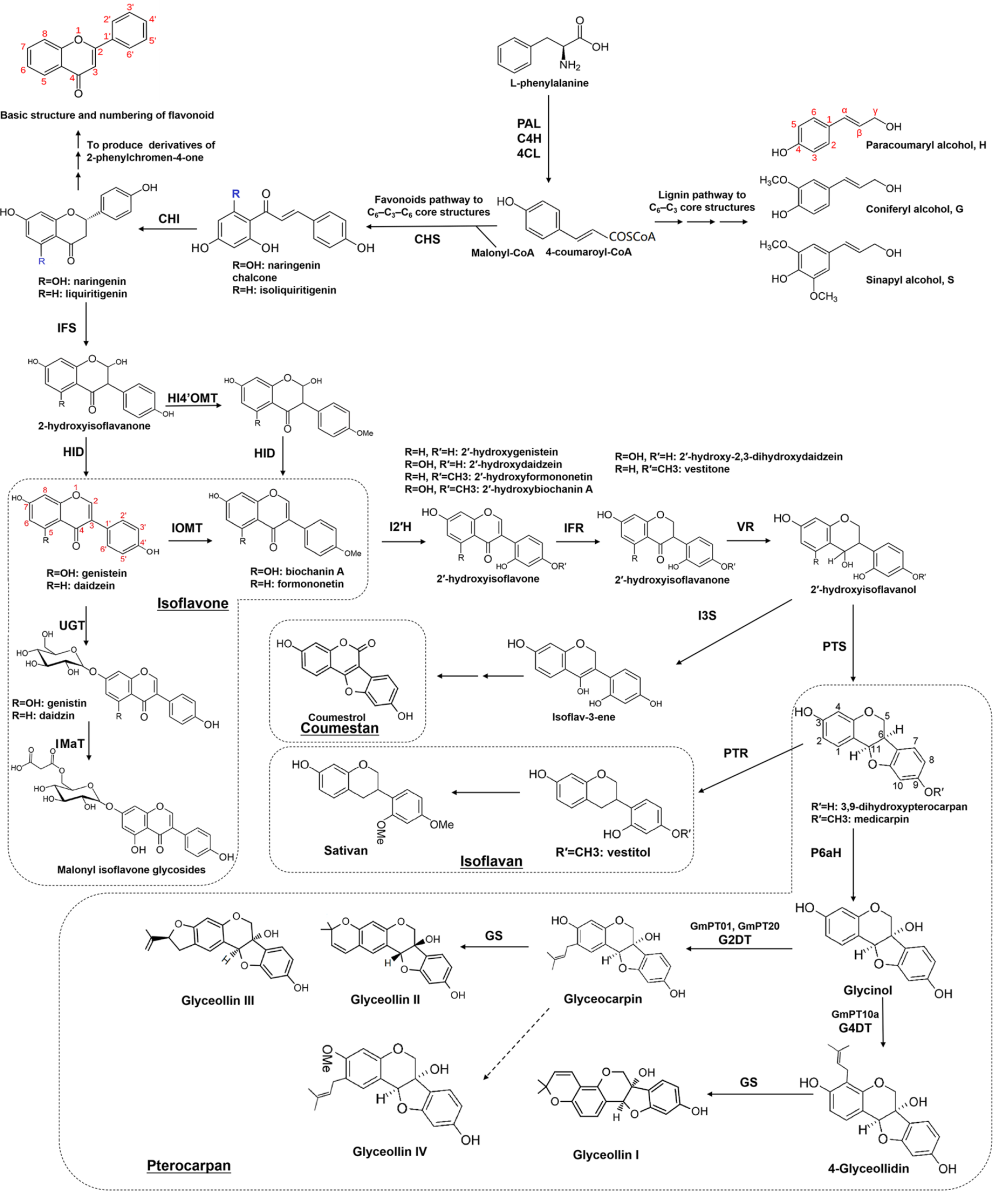
Biosynthetic pathways of isoflavonoids. PAL, phenylalanine ammonia-lyase; C4H, cinnamate 4-hydroxylase; 4CL, 4-coumarate CoA ligase; CHS, chalcone synthase; CHI, chalcone isomerase; IFS, isoflavone synthase; HI4′OMT, 2-hydroxyisoflavanone 4′-O-methyltransferase; HID/IFD, 2-hydroxyisoflavanone dehydratase; IOMT, isoflavonoid O-methyltransferase; UGT, glycosyltransferases; IMaT, isoflavone glucoside malonyltransferase; I2′H, isoflavone 2′-hydroxylase; IFR, isoflavone reductase; VR, vestitone reductase; PTS, pterocarpan synthase; PTR, Pterocarpan reductase; I3S, isoflav-3-ene synthase ; P6aH, pterocarpan 6a-hydroxylase; G2DT, glycinol 2-dimethylallyl transferase; G4DT, glycinol 4-dimethylallyl transferase; GS, glyceollin synthase.

Isoflavones, as shown in **Figure 2**, such as genistein, daidzein, biochanin A, formononetin, daidzin, ononin, etc., are found in various plants, with particularly high amounts in legumes like soybeans, chickpeas (*Cicer arietinum*), fava beans (*Vicia faba* L.), alfalfa (*Medicago sativa*), and medicinal plants like red clover (*Trifolium pratense*), *Glycyrrhiza uralensis*, and *Astragalus membranaceus* (Paiva et al., 1991; Kaufman et al., 1997; Tsao et al., 2006; Gao et al., 2015; Zhang et al., 2018). Almost all isoflavones exhibit antibacterial effects, aiding plants in resisting pathogens. Among them, genistein and daidzein serve as broad-spectrum antimicrobial agents, inhibiting the growth and reproduction of bacteria and fungi, thereby enhancing plant defense against pathogens (Dixon and Ferreira, 2002; Araya-Cloutier et al., 2017).

Pterocarpans, as shown in **Figure 2**, alongside isoflavones, are prevalent isoflavonoids found in various plants, including bitucarpin (A, B) and erybraedin C from *Bituminaria morisiana* and *Bituminaria. bituminosa*, medicarpin from *Medicago truncatula* and alfalfa, glycinol, and glyceollin from soybean, among others (Higgins, 1972; Paiva et al., 1991; Dixon, 1999; Pistelli et al., 2003; Naoumkina et al., 2007; Yoneyama et al., 2016; Sukumaran et al., 2018). In *M. truncatula*, medicarpin acts as an inducible “phytoalexin,” accumulating during defense responses (Naoumkina et al., 2007; Farag et al., 2008). In soybean, glycinol is formed through the cyclization of daidzein and serves as a precursor to glyceollin. Glyceollin is synthesized via the prenylation of glycinol in response to pathogen infection, enhancing resistance against soybean pathogens like *Phytophthora sojae*, *Diaporthe phaseolorum* var. *meridionales*, *Macrophomina phaseolina*, and *Sclerotinia sclerotiorum* (Akashi et al., 2009; Lygin et al., 2010; Yoneyama et al., 2016; Sukumaran et al., 2018). In *B. bituminosa*, the accumulation of pterocarpans (bitucarpin A and erybraedin C) significantly increases with treatment using arbuscular mycorrhizal fungi, offering a viable method for medicinal pterocarpan production (Pistelli et al., 2003, 2017).

Coumestrol, the principal coumestan compound, is derived from the conversion of unstable precursors isoflav-3-enes (**Figure 2**). It functions as both a phytoalexin and phytoestrogen, contributing to the well-being of both plants and humans (Martin and Dewick, 1980; Boue et al., 2000; Yuk et al., 2011; Ha et al., 2019; Uchida et al., 2020; Mun et al., 2021). For instance, the accumulation of coumestrol and the expression of genes involved in soybean coumestrol biosynthesis increase during leaf development, senescence, pathogen infection, nodulation, various stress treatments, and hormone treatments such as salicylic acid (SA), methyl jasmonate (MeJA), and ethylene (ET). This suggests that coumestans, including coumestrol, play crucial roles in plant development and stress defense (Boue et al., 2000; Lee et al., 2012; Tripathi et al., 2016; Ha et al., 2019; Mun et al., 2021; Ohta et al., 2021; Lee et al., 2022). Other coumestan compounds, like wedelolactone and psoralidin, exhibit similar functions. However, the key enzymes and regulators involved in their biosynthesis still require further exploration (Perez Rojo et al., 2023; Wang et al., 2023b).

Isoflavans, a specific subclass of isoflavonoids, are 5-deoxyisoflavonoids first discovered in legumes (**Figure 2**). This category includes glabridin from licorice (*Glycyrrhiza glabra*), vestitol, and sativan from *Lotus japonicus*, alfalfa, and others (Dewick and Martin, 1979; Hayashi et al., 1996; Shimada et al., 2000; Akashi et al., 2006; Veitch, 2009, 2013). The synthesis of vestitol in *Lotus* sp. is notably induced by ultraviolet (UV) radiation and the attachment of the root parasitic plant *Striga hermonthica*. Pterocarpan reductase (PTR) catalyzes this process, utilizing medicarpin as a substrate (Akashi et al., 2006; Ueda and Sugimoto, 2010; Kaducova et al., 2019; Kaducová et al., 2022). Subsequently, vestitol undergoes methylation by O-methyltransferase to produce sativan, another phytoalexin that accumulates significantly upon fungal pathogen infection in alfalfa and *Lotus* sp (Ingham and Harborne, 1976; Dewick and Martin, 1979; Saunders and O’neill, 2004; Trush et al., 2023). Glabridin, a prenylated isoflavan found in licorice plants, exhibits notable fungicidal activity against various phytopathogenic fungi such as *Fusarium graminearum*, *Sclerotinia sclerotiorum*, and *Corynospora cassiicola* (Simmler et al., 2013; Yang et al., 2021; Li et al., 2021a).

Rotenone and its derivative deguelin, the most common rotenoid compounds in *Derris* sp., are extensively employed as insecticides due to their capability to inhibit electron transport in the respiratory chain (Anzeveno, 1979; Hail and Lotan, 2004; Preston et al., 2017; Russell et al., 2020);. Additionally, rotenone exhibits anticancer activity *in vitro* but its application is limited due to neurotoxicity associated with its ability to cross the blood-brain barrier (Cannon et al., 2009; Deng et al., 2010). However, hydroxylated rotenoids are more hydrophilic and less likely to readily cross the blood-brain barrier, potentially acting as cell-selective killers to inhibit the proliferation of cancer cells (Naguib et al., 2018; Zhang et al., 2022a).

Indeed, the diversity and complexity of isoflavonoid compounds are largely due to chemical modifications such as methylation, glycosylation, and acylation. Differences in modification sites, types, and numbers of modifying groups can lead to changes in the physicochemical properties and biological activities of these derivatives. Therefore, studying the structure-function relationships of isoflavonoid derivatives and the related enzymes will contribute significantly to the efficient targeted synthesis of complex isoflavonoids in synthetic biology.

## Biosynthesis of isoflavonoids in plants

The biosynthesis of isoflavonoids begins with L‐phenylalanine and forms compounds with a classical C6-C3 core, which is called phenylpropanoid because of containing a benzene ring (phenyl) and a propionic acid side chain. The chemical intermediate p-coumaroyl-CoA catalyzed by 4-coumarate: CoA ligase (4CL) is a highly activated molecule, which acts as a key hub to determine the metabolic flow in response to developmental or environmental signals (**Figure 2**). One is to form molecules with a C6-C3 core structures, such as lignin monomers (including paracoumaryl alcohol, coniferyl alcohol, and sinapyl alcohol), and the other is to combine with Malonyl-CoA to form molecules with a C6-C3-C6 core, such as flavonoids (2-phenylchromen-4-one and derivatives) and isoflavonoids (3-phenylchromen-4-one and derivatives) (**Figure 2**).

Isoflavonoids can be divided into “phytoanticipins,” which are pre-existing compounds, including genistein, daidzein, formononetin, lupin, etc., or inducible “phytoalexins,” which are produced upon infection by pathogens or insects, such as medicarpin, pisatin, sativan, vestitol, glyceollin, and coumestrol (Dixon and Ferreira, 2002). While predominantly found in leguminous plants, isoflavonoids have been identified in various plant species beyond the legume family, including iridaceous, compositous, moraceous plants, barley (*Hordeum vulgare*), and others (Reynaud et al., 2005; Mackova et al., 2006; Darbour et al., 2007; Abderamane et al., 2011; Picmanov et al., 2012; Polturak et al., 2023). A recent study in wheat unveiled a pathogen-induced biosynthetic gene cluster with seven enzymes, including chalcone synthase (CHS1), non-induced chalcone isomerase (CHI), two cytochrome P450s, and three O-methyltransferases (OMTs), resulting in the production of a wheat-specific isoflavonoid named triticein (Polturak et al., 2023). This breakthrough provides new opportunities for isoflavonoid synthesis in non-legume crop plants.

As shown in **Figure 2**, the isoflavonoid biosynthetic pathway initiates with isoflavone synthase (IFS), catalyzing the migration of the B-ring from the 2- to the 3-position of the C ring, yielding 2-hydroxyisoflavanone (Jung et al., 2000; Subramanian et al., 2006). Subsequent dehydration by 2‐hydroxyisoflavanone dehydratase (HID/IFD) results in isoflavones like genistein and daidzein (Hakamatsuka et al., 1998; Akashi et al., 2005; Shimamura et al., 2007). In soybeans (*Glycine max*), another HID/IFD converts 4′-methoxylated 2-hydroxyisoflavanones into formononetin or biochanin A (Akashi et al., 2005). Diverse isoflavonoid compounds result from multiple O-methyltransferases (OMTs) methylating at the C3′-,4′, 3-, or 7-hydroxyl group. Various derivatives, such as 3’-methoxy-puerarin, 4′-O-methylated isoflavonoid formononetin, and 7-O-methylated isoflavone isoformononetin, are formed (He and Dixon, 2000; Liu and Dixon, 2001; Zubieta et al., 2001; Akashi et al., 2003; Liu et al., 2006; Li et al., 2016a). Subsequently, methylated formononetin transforms into medicarpin through isoflavone reductase (IFR), vestitone reductase (VR), and pterocarpan synthase (PTS) (Paiva et al., 1991; Oommen et al., 1994; Dixon et al., 1995; Guo and Paiva, 1995; Nakamura et al., 1999; Uchida et al., 2017). Glycosyltransferases (UGTs) further contribute to derivatives synthesis, e.g., 7-O-glucosyltransferase converting daidzein to daidzin and 8-C-glucosyltransferase forming puerarin from daidzein (He and Dixon, 2000; Noguchi et al., 2007; Li et al., 2014; Funaki et al., 2015; Wang et al., 2017). Acyltransferases add acyl groups (prenyl or acetyl) to produce prenylated or acetylated isoflavonoids, enhancing antimicrobial activities (Dixon, 1999; Mukne et al., 2011; Shen et al., 2012; Ahmad et al., 2017; Araya-Cloutier et al., 2017; Mouffouk et al., 2017; Araya-Cloutier et al., 2018; Kalli et al., 2021).

**Figure 3:**
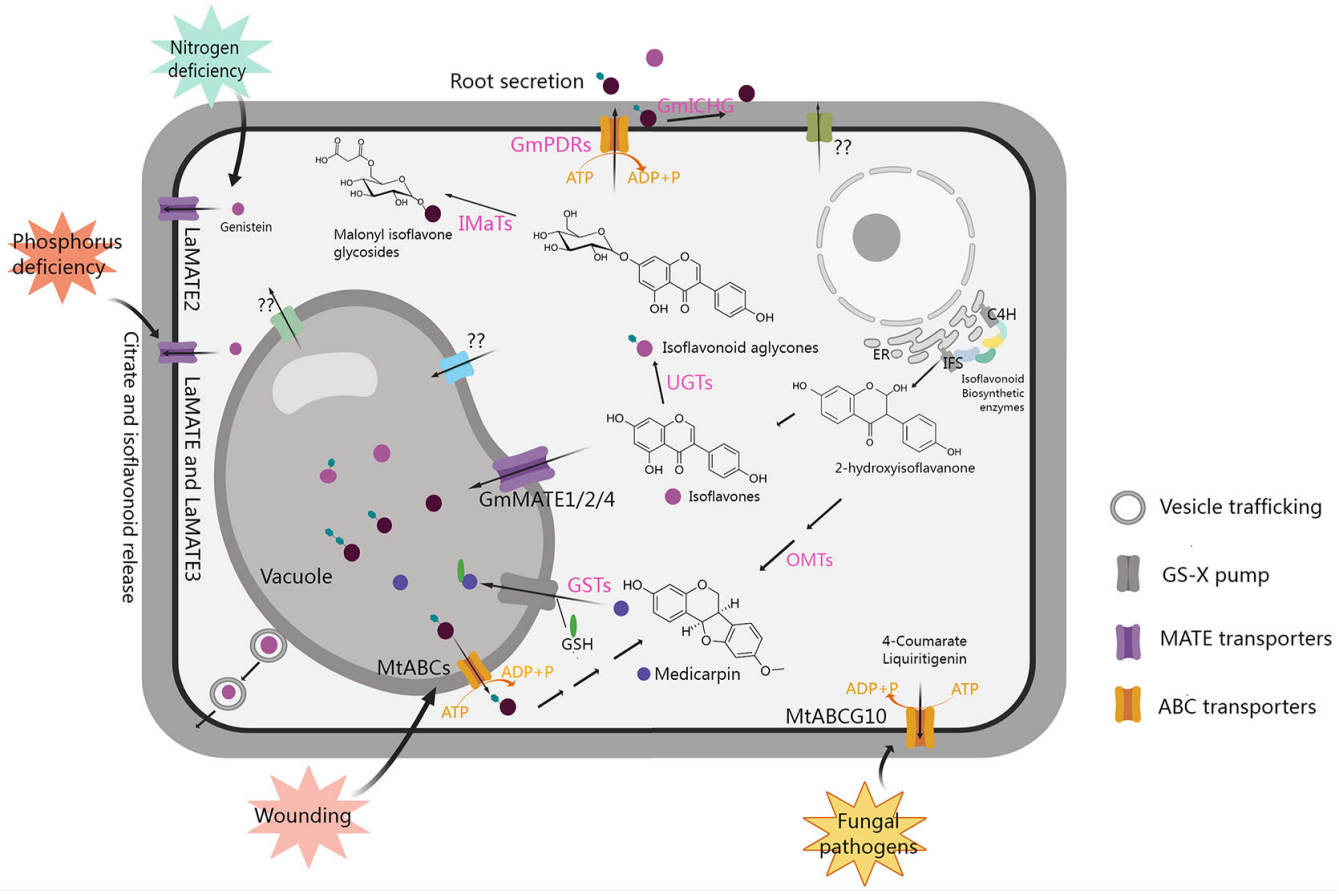
Transport and accumulation of isoflavonoids in legumes. GS-X pump, glutathione conjugates pump; PDRs, pleiotropic drug resistance transporters belonging to the ABCG subfamily; GSTs, glutathione S-transferases; ER, endoplasmic reticulum. The figure was created with Medpeer (https://image.medpeer.cn/).

A large number of isoflavonoid compounds are produced in plants during specific developmental stages or in response to environmental signals, which are accompanied by re-distribution of substrates and induced phytoalexin synthesis (Piślewska et al., 2002; Gutierrez-Gonzalez et al., 2009, 2010). In many times, the encoding genes of these enzymes only function after being induced by speific factors (Shimada et al., 2007; Shelton et al., 2012). Therefore, key enzymes in the isoflavonoid biosynthetic pathway, especially those involved in the modification of isoflavonoids such as glycosyltransferases and methyltransferases for the modification of -OH groups at C-7, C-5, or C-4’, glycosidases for hydrolysis glycosylated isoflavonoids, and acyltransferase for malonylation or acetylation of isoflavonoids are still not fully characterized, and their functions require further elucidation (**Figures 2**, **3**). To comprehensively explore these biosynthetic pathways and regulatory mechanisms, germplasm resources of legumes can be collected or established, and integrated approaches such as genome resequencing techniques, transcriptomics, and metabolomics can be applied to identify the genes responsible for synthesizing key enzymes.

## Transport of isoflavonoids in legumes

Extensive studies have indicated that glycosylation and acylation modifications of isoflavonoids not only enhance transporter affinity and efficiency, but also improve water solubility and stability, facilitating storage in vacuoles or extracellular secretion (Dixon, 1999; Zhao et al., 2011; Le Roy et al., 2016; Ahmad et al., 2017; Ku et al., 2020; Ahmad et al., 2021). Additionally, isoflavonoids can act as signaling molecules by translocating to the nucleus and influencing the expression of downstream genes (Naoumkina et al., 2007; Naoumkina and Dixon, 2008; Yu et al., 2008; Zhao and Dixon, 2010). As shown in **Figure 3**, various transport mechanisms, including vesicle trafficking, ATP-binding cassette (ABC) transporters, multidrug and toxic compound extrusion (MATE) transporters, and glutathione S-transferase (GST), located in the vacuolar membrane or plasma membrane, have been reported to play roles in the transport and distribution of isoflavonoids (Li et al., 1997; Naoumkina et al., 2007; Naoumkina and Dixon, 2008; Zhao and Dixon, 2010; Zhao et al., 2011; Banasiak et al., 2013; Zhao, 2015; Biala-Leonhard et al., 2021).

In leguminous plants, isoflavonoids released by the roots serve as signaling molecules, influencing rhizobia to promote symbiosis. Identified transporters play crucial roles in the release of isoflavonoids from roots. For instance, in *Lupinus albus*, phosphorus deficiency induces the expression of *LaMATE*, *LaMATE2*, and *LaMATE3*, as well as the release of genistein from roots. The individual silencing of these genes reduces genistein release. Among them, LaMATE2 has been confirmed to transport genistein using yeast microsomal membrane vesicles, while the isoflavone transport functions of LaMATE and LaMATE3 require further exploration (**Figure 3**). Additionally, *LaMATE2* is induced by nitrogen deficiency, and its silencing also reduces the number of nodules, indicating that LaMATE2 facilitates the export of isoflavonoids into the rhizosphere, fostering nodule formation (Biala-Leonhard et al., 2021; Zhou et al., 2021). In *M. truncatula*, a plasma membrane-localized ABC transporter MtABCG10, which belongs to the G subfamily, is involved in medicarpin precursor transport, modulates the distribution of 4-coumarate and liquiritigenin during nodule formation and plant defense against fungal pathogens (Jasinski et al., 2009; Banasiak et al., 2013; Biala et al., 2017). LjABCG1, which is homologous to the MtABCG10 in *L. japonicus*, is associated with pathogenesis rather than symbiosis, but the substrate remains unclear and requires further investigation (Sugiyama et al., 2015). In soybean, 13 pleiotropic drug resistance genes (*PDRs*) encoding ABCG transporters were found to be expressed in roots. One of them was confirmed to participate in membrane transport of genistein, daidzein, and other isoflavonoid aglycones, initiating legume-rhizobium symbiosis formation (Sugiyama et al., 2007, 2008). Additionally, the β-glucosidase GmICHG in soybean cell walls hydrolyzes genistin, contributing to isoflavone secretion in roots (Suzuki et al., 2006).

Transporters also play a crucial role in directing isoflavonoids into the vacuole for storage. In soybean, GmMATE1, GmMATE2, and GmMATE4, localized in the vacuolar membrane, are implicated in transporting isoflavones for accumulation through yeast uptake assay. In addition, it has also been confirmed that the total isoflavone content in seeds is significantly increased due to the overexpression of *GmMATE1* in transgenic soybean, while it is significantly decreased due to *GmMATE1* mutation (Ng et al., 2021; Ku et al., 2022). The utilization of maize GST-mediated conjugation with GSH (medicarpin-GS) significantly enhances the uptake of isoflavonoids by vacuoles (Li et al., 1997). When plants are exposed to stress-induced signals, they utilize store isoflavonoids to synthesize phytoalexin to enhance their resistance. For example, wound signal MeJA induces a decrease in the content of isoflavone glycosides, while it can also induce the accumulation of medicarpin in alfalfa, which is accompanied by the up-regulation the expression of multiple ABC transporter genes and β-glucosidase genes, suggesting that ABC transporters may be involved in transport of isoflavone glycosides from the vacuole to the cytoplasm for the synthesis of medicarpin, thereby increasing plant resistance to wounding (Naoumkina et al., 2007). However, the specific ABC transporters involved in this process remain unclear and require further exploration.

As shown in **Figure 3**, the transporters that have been reported so far are mainly involved in the transport of isoflavones such as genistein, while there is scarce research on proteins that transport other types of isoflavonoids. Among them, although MtABCs may be involved in the transport of medicarpin, it is still unclear which ABC protein is responsible. There are also some transporter proteins whose encoding gene expression levels can affect the change in isoflavone content, but whether they have a direct transport function needs further confirmation. Overall, a more in-depth understanding of the transporters involved in their conveyance, accumulation, and extracellular secretion is essential for unraveling their physiological and pathological actions in plants.

## Regulation of isoflavonoid biosynthesis

The biosynthesis of isoflavonoids in plants is intricately regulated by diverse environmental factors (such as UV radiation, fungal infection, nitrogen, and phosphorus deficiencies), through transcriptional regulation, post-translational modifications, and epigenetic changes (Dastmalchi et al., 2017; Su et al., 2021; Wang et al., 2023a).

Numerous studies have demonstrated the accumulation of isoflavonoid in plants is also induced by hormonal signals (Ng et al., 2015; Boivin et al., 2016; Yuk et al., 2016; Jeong et al., 2018; Kurepa et al., 2023). The dynamic presence or absence of isoflavonoids significantly influences plant resistance to fungi and environmental stresses, shaping plant growth and development through intricate modulation of auxin transport *in vivo* (Mathesius, 2001; Wasson et al., 2006; Gao et al., 2021; Zhang et al., 2022b). For instance, in alfalfa, exposure to a fungal elicitor promptly triggers the accumulation of the phytoalexin medicarpin, subsequently undergoing glycosylation and malonylation to form isoflavonoid conjugates (Kessmann et al., 1990). In *M. truncatula*, fungal infection triggers *de novo* medicarpin biosynthesis, while wound signals induce the downstream genes converting formononetin and isoflavone glycosides into medicarpin (Naoumkina et al., 2007; Farag et al., 2008). RNA interference-induced silencing of chalcone synthase gene (*CHS*) in *M. truncatula* amplifies auxin transport, leading to an impaired ability to form nodules and a deficiency in (iso)flavonoids, particularly formononetin, daidzein and medicarpin (Wasson et al., 2006). When using these compounds and their glycoside forms to treat the wild-type roots, only the free formononetin significantly inhibited auxin transport. However, compared to the wild type, *CHS* silencing increased auxin transport in roots, indicating that isoflavonoid acts as an auxin transport inhibitor in *M. truncatula* (Laffont et al., 2010). Additionally, transcriptome analysis of *M. truncatula* root hairs during rhizobial infection reveals the induction of genes associated with auxin signaling, strigolactone (SL), gibberellic acid (GA), brassinosteroid (BR), and medicarpin biosynthesis, accompanied by the repression of genes involved in lignin biosynthesis. This emphasizes the pivotal roles of (iso) flavonoids and plant hormones, particularly auxin, in the context of rhizobial infection (Breakspear et al., 2014).

An optimal auxin gradient is required for the formation and development of legume nodule primordia (Benkova et al., 2003; Kohlen et al., 2018). In soybean, the involvement of the PIN-FORMED auxin transporter GmPIN1 in nodulation has been elucidated. This process is mediated by two nodulation regulators, (iso)flavonoids (genistein and 7,4′-dihydroxyflavone), which expand GmPIN1 distribution, and cytokinin, which rearranges the cellular polarity of GmPIN1. This orchestration establishes an appropriate auxin gradient, fostering soybean nodulation (Gao et al., 2021). Furthermore, GmPIN1 is involved in the polar transport of auxin from the leaf to the petiole base, resulting in an asymmetric distribution of auxin in the upper and lower petiole cells. This asymmetry significantly impacts cell expansion and the leaf petiole angle. Light-induced (iso)flavonoids accumulate more in the upper petiole cells, inhibiting GmPIN1 expression and disrupting its distribution, leading to reduced auxin in the upper petiole cells. Conversely, lower petiole cells, with lower (iso)flavonoid levels, accumulate more auxin, promoting cell expansion (Zhang et al., 2022b). In addition, cytokinin signaling induces the expression of (iso)flavonoid synthesis genes, influencing the accumulation of (iso)flavonoids and auxin transport, thereby impacting nodule formation (Goyal and Ramawat, 2008; Ng et al., 2015).

**Figure 4:**
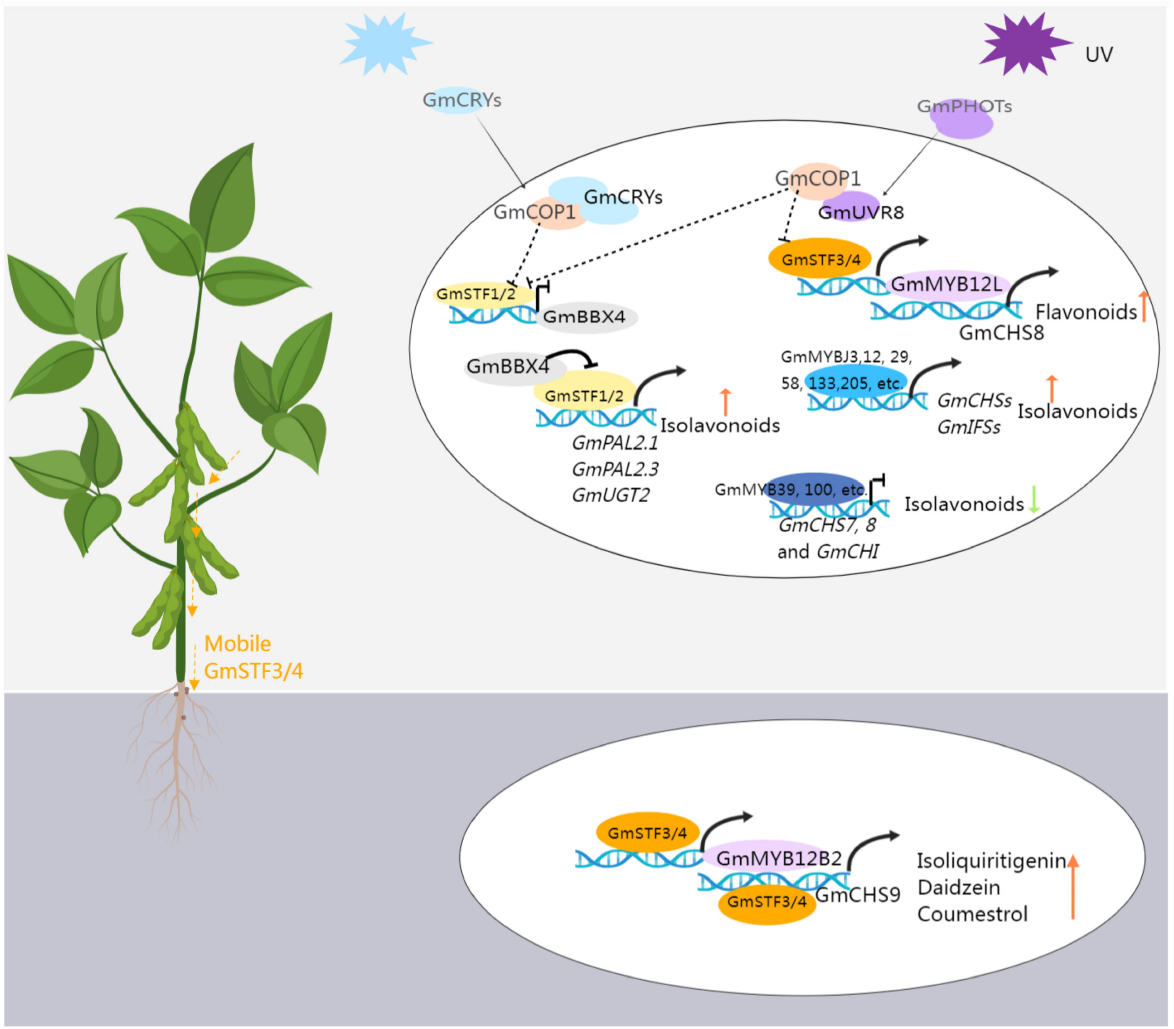
The transcriptional regulation pattern of isoflavonoid biosynthesis in soybean. Blue light activates the GmCRVs receptors, which interacts with GmCOP1, releasing GmSTF1/2 from the STF-COP1 complex. This activation leads to the expression of *GmPAL2* and *GmUGT2* by GmSTF1/2, promoting the accumulation of isoflavonoids. Additionally, at the transcriptional level, GmSTF1/2 can inhibit the expression of *GmBBX4*. At the protein level, GmBBX4 interacts with GmSTFs and suppresses their ability to activate target genes related to isoflavonoid synthesis, creating a negative feedback loop. UV activation of the URV receptor allows it to interact with COP1, releasing STF3/4 from the STF-COP1 complex. STF3/4 then activates *GmMYB12L*, which in turn activates the expression of *GmCHS8*, promoting the synthesis of flavonoids. Moreover, STF3/4 can move from the shoot to the soybean root, activating the expression of *GmMYB12B2* and *GmCHS9*, promoting the synthesis of (iso)flavonoid. The figure was created with Medpeer (https://image.medpeer.cn/).

As previously discussed, (iso)flavonoid accumulation is induced in response to light or UV radiation, a process mediated by proteins engaged in light signal transduction pathways. As shown in **Figure 4**, these include UV-A and blue-light photoreceptors cryptochromes (CRYs) and phototropins (PHOTs), along with CONSTITUTIVELY PHOTOMORPHOGENIC 1 (COP1, an E3 ubiquitin ligase), ELONGATED HYPOCOTYL 5 (HY5, a basic leucine-zipper transcription factor), and B-BOX CONTAINING PROTEINs (BBXs) (Xu, 2020; Liu et al., 2023). A recent study confirmed that the photoreceptors–COP1–HY5-BBX4 regulatory module could regulate the isoflavonoid biosynthesis in soybean (Song et al., 2023). GmSTF1 and GmSTF2 (HY5 orthologs) serve as positive regulators of isoflavonoid synthesis, activating the expression of *GmPAL2.1*, *GmPAL2.3*, and *GmUGT2* while repressing *GmBBX4* expression. GmBBX4, in turn, inhibits the transcriptional activation activity of GmSTF1/2 through direct interaction. Photoreceptors CRYs and PHOTs play positive regulatory roles in light-signal-mediated isoflavonoid biosynthesis. Conversely, COP1, acting as their genetically downstream component, negatively regulates isoflavonoid synthesis by promoting the degradation of GmSTF1/2. Furthermore, GmSTF3/4 are involved in UV-mediated isoflavonoid synthesis, responding to UV-B light through the UV-B photoreceptor (UVR8) in shoots. This signal is then transmitted to the roots, activating the expression of *GmMYB12B2* and *GmCHS9*, subsequently increasing the isoflavone content (Chen et al., 2024). Investigating the impact of light on isoflavone accumulation provides both a theoretical basis and technical support for intercropping. For instance, in maize-soybean intercropping, shading effects from maize growth, limiting photosynthesis, were found to decrease mildew incidence on soybean pods, attributed to the accumulation of isoflavones in soybean under shading conditions, particularly during the vegetative stage (Li et al., 2021b).

Key enzymes in isoflavonoid biosynthesis, such as CHS, CHI, and IFS, are regulated by specific transcription factors (TFs). Notably, MYB TFs have emerged as direct regulators of isoflavonoid biosynthesis genes. As shown in **Table 1**, GmMYB29, GmMYB58, GmMYB205, GmMYBJ3, GmMYB12, GmMYB133, and GmMYB502 act as activators to increase the expression of *CHS* and *IFS* genes, thereby promoting the accumulation of isoflavonoids in soybean, while GmMYB39 and GmMYB100 are repressors. It is worth mentioning that GmMYB176, an R1 MYB TF, plays a dual role in isoflavonoid biosynthesis. And it decreases the isoflavonoids accumulation by down-regulating the expression of GmIFS, and increases the flavanone liquiritigenin content by activating the expression of GmCHS8, respectively. In the presence of GmbZIP5, however, GmMYB176 acts as a positive regulator to enhance the accumulation of some other isoflavonoids, such as glyceollin, isowighteone and a unique O-methylhydroxy isoflavone (Anguraj Vadivel et al., 2019, 2021). Besides, the intracellular localization of GmMYB176 or GmMYB133 can be modulated through interactions with 14–3-3 proteins, such as GmSGF14. This interaction subsequently hinders their regulatory role in isoflavonoid biosynthesis (Li and Dhaubhadel, 2012; Li et al., 2012; Bian et al., 2018). In other plant species, various MYB TFs have been documented, including positive regulators LjMYB14 and LjMYB36 in *L. japonicus*, CaMYB39 in chickpea, and a negative regulator MtPAR in *M. truncatula* (**Table 1**). MtPAR serves as a switch for proanthocyanidin synthesis, capable of directly inhibiting the expression of *IFS2*. Conversely, it activates the expression of anthocyanidin reductase (ANR) encoding gene in the presence of MtTT8 and MtWD40–1, leading to a reduction in isoflavone and anthocyanidin levels, thereby channeling metabolic flux towards proanthocyanidin biosynthesis (Verdier et al., 2012; Li et al., 2016b, c).

**Table 1:** Regulators involved in isoflavonoid biosynthesis.

Regulators		Regulatory effect	Target genes/proteins	References
Category	Name			
**R2R3 MYB TF**	GmMYB29	Activator	*GmIFS2* and *GmCHS8*	(Chu et al., 2017)
	GmMYB29A2	Activator	*IFS2* and *G4DT*	(Jahan et al., 2020)
	GmMYB58 and GmMYB205	Activator	*GmCHS*, *GmIFS2*, and *GmHID*	(Han et al., 2017)
	GmMYBJ3	Activator	*GmCHS8* and *GmCHI1A*	(Zhao et al., 2017)
	GmMYB1	Activator	*GmCHS8* and *GmIFS2*	(Bian et al., 2018)
	GmMYB502	Activator	*GmCHS8*, *GmIFS1*, and *GmIFS2*	(Sarkar et al., 2019)
	GmMYB12	Activator	*GmCHS8* and *GmCHS9*	(Chen et al., 2024)
	GmMYB39	Repressor	*GmCHS8*	(Liu et al., 2013)
	GmMYB100	Repressor	*Gm*CHS7 and *G*mCHI	(Yan et al., 2015)
	MtPAR	Inhibitor	*IFS2* and *ANR*	(Li et al., 2016c)
	LjMYB14	Activator	It is likely to be *IFS* and *IFR*.	(Shelton et al., 2012)
	LjMYB36	Activator	Unconfirmed	(Monje-Rueda et al., 2023)
	AtMYB12	Activator	*GmIFS1*	(Pandey et al., 2014)
	CaMYB39	Activator	*CHS*, *CHI*, *F3H*, F3’ H, and FLS.	(Saxena et al., 2023)
**R1 MYB TF**	GmMYB176	Dual functions	Activate *GmCHS8* but down-regulate *GmIFS*, while interact with GmbZIP5 to enhance the level of isoflavonoids	(Yi et al., 2010; Anguraj Vadivel et al., 2019, 2021)
**14–3-3 protein**	GmSGF14	A negative regulator	Inhibit the function of GmMYB176.	(Li and Dhaubhadel, 2012; Li et al., 2012)
**NAC**	GmNAC42–1	Activator	*IFS2* and *G4DT*	(Jahan et al., 2019)
**C2H2-type zinc-finger**	GmZFP7	Activator	*GmIFS2* and *GmF3H1*	(Feng et al., 2023)
**E3 ubiquitin ligase**	GmCOP1b	A negative regulator	Promote the degradation of GmSTF1/2.	(Song et al., 2023; Chen et al., 2024)
**HY5**	GmSTF1/2	Activator	*GmPAL2.1*, *GmPAL2.3*, *GmUGT2* and *GmBBX4*	
	GmSTF3/4	Activator	*GmMYB12L*, *GmMYB12B2*, and *GmCHS9*	
**B-BOX PROTEIN**	GmBBX4	A negative regulator	Inhibit the transcriptional activation activity of GmSTF1 and GmSTF2.	
**MicroRNA**	*Gma-miRNA393*	A positive regulator	Unconfirmed	(Wong et al., 2014)
	*Gma-miRNA5030*	A negative regulator	It is likely to be *GmMYB176*.	(Gupta et al., 2019b)
	*Gma-miRNA* *12/24/29*	A negative regulator	Correspond to *Glyma.08G181000, Glyma.10G224000*, and *Glyma.02G279600*, encoding different UGTs	(Gupta et al., 2017)
	*Gma-miRNA26*	A negative regulator	It is likely to be *Glyma.10G197900*, encoding a 4-coumarate-CoA ligase	(Gupta et al., 2019a; Elseehy, 2020)
	*Gma-miRNA28*	A negative regulator	It is likely to be *Glyma.09G127200*, encoding an isoflavone 7-OMT.	
DNA methyltransferase	Unconfirmed	Cytosine methylation	IFS genes	(Gupta et al., 2019a; Elseehy, 2020)

Additional regulators, including NAC and C2H2-type zinc-finger TFs, are also implicated in isoflavonoid biosynthesis. For instance, the expression of *GmNAC42–1* responds to both abiotic and biotic elicitors, stimulating the synthesis of pterocarpan glyceollin by activating *IFS2* and *G4DT* (encoding glycinol 4-dimethylallyl transferase) in soybean (Jahan et al., 2019). Notably, GmNAC42–1 is under positive regulation by GmMYB29A2, which itself acts as a positive regulator in the glyceollin biosynthetic pathway (Jahan et al., 2020). Another player, GmZFP7, a C2H2 zinc-finger TF, has been reported to modulate isoflavone accumulation by activating *GmIFS2* and *Flavanone 3 β-hydroxylase 1* (*GmF3H1*) in soybean (Feng et al., 2023).

Recent studies also reveal the involvement of microRNAs (miRNAs) in post-transcriptional regulation of isoflavonoid biosynthesis. In soybean, *P. sojae* infection induced the expression of *Gma-miRNA393* in roots. Knockdown of *Gma-miRNA393* reduced isoflavonoid content and downregulated the gene expression of *GmHID1* and *GmIFS1*, while increasing susceptibility to *P. sojae*. This suggests that Gma-miRNA393 acts as a positive regulator of isoflavonoid biosynthesis; however, its downstream target genes remain unidentified (Wong et al., 2014). **Table 1** delineates additional miRNAs, including Gma-miRNA12, Gma-miRNA24, Gma-miRNA29, Gma-miRNA26, and Gma-miRNA28, acting as negative regulators by interfering with the expression of their target genes. These target genes encode key enzymes or transcription factors crucial in isoflavonoid biosynthesis (Gupta et al., 2017; Gupta et al., 2019b).

Moreover, epigenetic regulation, including DNA methylation and histone modifications, has been implicated in the control of isoflavonoid accumulation (Baulcombe and Dean, 2014; Chang et al., 2020). A comprehensive comparative analysis across various soybean genotypes exhibiting distinct isoflavone contents revealed a positive correlation between the expression of the *IFS* gene and the cytosine methylation level within its coding region (Gupta et al., 2019a). This finding underscores the potential positive regulatory impact of epigenetic modifications on the intricate process of isoflavonoid biosynthesis. In the first generation (T1) of transgenic wheat (*Triticum aestivum*) overexpressing *IFS*, methylation levels in the exogenous promoter region exhibited a negative correlation with *IFS* expression (Elseehy, 2020), implying that T1 plants can reconstitute gene expression by altering the methylation status of the exogenous promoter.

Collectively, as shown in **Figure 4** and **Table 1**, although many genes involved in the regulation of isoflavonoid synthesis and their target genes have been reported, some regulatory mechanisms are still unclear. These regulatory genes often affect the synthesis of multiple isoflavonoid compounds simultaneously. Therefore, in future, more specific factors need to be explored, such as those that uniquely control the synthesis of the subclass of isoflavonoids. In addition, as mentioned earlier, environmental factors and hormone signals play a significant role in isoflavonoid synthesis, regulation, and transport, which is also an important direction for future exploration.

## Metabolic engineering of isoflavonoids biosynthesis

Isoflavonoids, recognized for their benefits in plants, livestock, and human health, have spurred research in metabolic engineering. As shown in **Table 2**, strategies like antisense RNA, RNA interference, CRISPR/Cas9-mediated gene editing, co-expression, and heterologous expression have been employed for isoflavonoid engineering in legumes, non-legume plants, and microorganisms (Dixon and Steele, 1999; Zhang et al., 2020; Liu et al., 2021).

**Table 2:** Metabolic engineering of isoflavonoid biosynthesis in model plants and microbial hosts.

Strategy	Target genes	Results	Species	References
**Overexpression**	*GmMYB176* and *GmbZIP5*	Accumulation of multiple isoflavonoids	Soybean	(Anguraj Vadivel et al., 2021)
**Antisense RNA**	*CCoAOMT*	Accumulation of medicarpin upon fungi infection	Alfalfa	(Gill et al., 2018)
**RNA interference**	*GmFNSII-1* and *GmFNSII-2*	Accumulation of isoflavone	Soybean	(Jiang et al., 2010)
	*GmF3H* and *GmFNSII*	Accumulation of isoflavone	Soybean	(Jiang et al., 2014)
**Heterologous expression**	*GmIFS*	Accumulation of genistein	Arabidopsis mutant (tt6/tt3)	(Liu et al., 2002)
	Co-verexpression of *CRC* and *F3H*	Accumulation of total isoflavone	Soybean	(Yu et al., 2003)
	*MtIFS1*	Accumulation of isoflavonoid	Alfalfa	(Deavours and Dixon, 2005)
	Fusion of *GmIFS2* and alfalfa *CHI*	Accumulation of isoflavonoid	Yeast and tobacco	(Tian and Dixon, 2006)
	*GmIFS*	Accumulation of genistein	Transgenic tobacco with antisense of F3H	(Liu et al., 2007)
	*GmIFS*	Accumulation of genistein derivatives	Rice	(Sreevidya et al., 2006)
	*GmIFS2*	Accumulation of genistin	Tomato	(Shih et al., 2008)
	*GmIFS2*	Accumulation of genistein derivatives	Brassica napus	(Li et al., 2011)
	*AtMYB12* and *GmIFS1*	Accumulation of genistein glycoconjugates	Tobacco	(Pandey et al., 2014)
	*GmIFS1*, *GmCHS7* and *GmCHI1*	Both isoflavone and proanthocyanidin accumulation	M. truncatula	(Gou et al., 2016)
	*GmCHIs* and *GmIFS*	Transformation of chalcones into isoflavonoids	Yeast	(Ralston et al., 2005)
CRISPR/Cas9-mediated gene-editing	*GmF3H1*, *GmF3H2* and *GmFNSII-1*	Improvement of isoflavone content and resistance to mosaic virus	Soybean	(Zhang et al., 2020)
De novo biosynthesis	*At4CL1*, *GmCHR5*, *GmCHS8*, *GmCHI1B2*, *Ge2-HIS*, *GmHID* and *GmUGT4*	De novo biosynthesis of isoflavonoids	Yeast	(Liu et al., 2021)

The main strategies for forage legumes breeding to increase the nutritional value and digestibility of forage include reducing anti-nutritional factors, such as lectins, saponins, oxalic acid, and condensed tannins, increasing crude protein concentrations, enhancing stress tolerance, and changing cell wall structure and composition to improve the degradability of cell wall polysaccharides (Kumar, 2011; Kulkarni et al., 2018; Katoch, 2022). Interestingly, most of these breeding goals can be achieved through metabolic engineering of phenylpropanoid biosynthesis (Dixon and Steele, 1999; Du et al., 2010). In alfalfa, overexpression of the encoding gene of isoflavone O-methyltransferase (IOMT) results in heightened levels of formononetin and medicarpin, enhancing disease resistance to *Phoma medicaginis* in transgenic plants (He and Dixon, 2000). Heterologous expression of *MtIFS1* can lead to the enhanced accumulation of medicarpin in transgenic alfalfa plants upon *P. medicaginis* infection, indicating that this modification is beneficial for plant response to stress (Deavours and Dixon, 2005). Moreover, Gou et al. disrupted the limitation of precursors by simultaneously overexpressing of *GmIFS1*, *GmCHS7* and *GmCHI1* in *M. truncatula*, promoted both isoflavone and proanthocyanidin accumulation, which are beneficial for ruminant animals (Gou et al., 2016). As shown in **Table 2**, the key enzyme genes involved in isoflavonoid biosynthesis are important target genes for metabolic engineering, *Caffeoyl-CoA O-methyltransferase* (*CCoAOMT*) encoding a key enzyme of lignin pathway also has an important impact on isoflavonoid synthesis. Down-regulated of *CCoAOMT* via antisense RNA technology, leading to a decrease in the content of guaiacyl (G) lignin and an increase in syringyl to guaiacyl ratio (S/G) (Guo et al., 2001). When wild-type and *CCoAOMT* downregulated plants are infected with fungi, the expression of medicarpin biosynthesis genes is upregulated in both, but more significantly in the *CCoAOMT* downregulated plants. This leads the lignin modified alfalfa to redirect metabolic flux towards the medicarpin pathway upon fungal infection, thereby improving the availability of cell wall polysaccharides and resistance against fungal disease (Guo et al., 2001; Gill et al., 2018).

In soybean, the overexpression of *F3H* alone does not significantly affect the total isoflavone content. However, the overexpression of a fusion gene of the maize *C1* and *R* (*CRC*) increases the total isoflavone content in transgenic soybean seeds by approximately 2-fold. Co-verexpression of *CRC* and *F3H* can enhance the total isoflavone content by about 4-fold (Yu et al., 2003). RNA interference was used to generate silence *FNSs* (encoding flavone synthases) in soybean, which reduced the synthesis of apigenins and anthocyanins from naringenin, thus promoting the accumulation of isoflavones (Jiang et al., 2010). Notably, silencing alone of *FNSII* or *F3H* results in a ~1.3- or ~1.9-fold increase in isoflavone content, while double silencing of *FNSII* and *F3H* can result in a ~2.2-fold increase in isoflavone production compared to transgenic soybean hairy roots containing empty vectors (Jiang et al., 2014). Co-overexpression of *GmMYB176* and *GmbZIP5* results in an approximate 1.4-fold increase in the total isoflavonoid content in hairy roots (Anguraj Vadivel et al., 2021).

Non-legume plants and microorganisms can also synthesize large amounts of isoflavonoids through metabolic engineering, which involves utilizing the existing flavonoid biosynthesis pathway to provide precursors and introducing the key enzyme genes for isoflavonoid biosynthesis. For example, heterologous expression *GmIFS* in *Arabidopsis thaliana* could accumulate a small amount of genistein glycosides while introducing the GmIFS gene into *tt6/tt3* double mutant, where expression of *F3H* and *dihydroflavonol reductase* (*DFR*) was abolished, resulted in a large accumulation of genistein, which provides an important idea for the isoflavonoid accumulation via metabolic engineering (Liu et al., 2002). Heterologous expression of the IFS/CHI fusion gene in tobacco results in higher levels of genistein and its glycoside compounds compared to expressing IFS alone (Tian and Dixon, 2006). When *AtMYB12* and *GmIFS1* are co-overexpressed in tobacco, the expression of key enzyme genes in the flavonoid pathway is significantly upregulated, leading to a substantial increase in the content of flavonoid compounds and synthesizing approximately 0.05 mg/g of genistein in the fresh tissues (Pandey et al., 2014). In addition, isoflavonoids could be synthesized and accumulated in non-legume plants through the heterologous expression of *GmIFSs* in rice, tomato and *Brassica napus* (Sreevidya et al., 2006; Tian and Dixon, 2006; Liu et al., 2007; Shih et al., 2008; Li et al., 2011; Pandey et al., 2014). These results indicate that heterologous expression of key enzyme genes can achieve the synthesis of isoflavones in non-leguminous plants. However, to achieve high content, a strategy of co-expressing multiple structural genes or a combination of transcription factors with structural genes can be employed. Additionally, the activity of key isoflavone biosynthetic enzymes may vary in different non-leguminous plants, which may potentially affect the yields. For example, CHIs are divided into two groups (type I and type II). Type I CHIs, which are found in both legumes and non-legumes, function to isomerize only 6′-hydroxychalcone to 5-hydroxyflavanone (naringenin). Whereas, type II CHIs belong to a legume-specific group that are active on both 6′-deoxychalcone and 6′-hydroxychalcone, yielding 5-deoxyflavanone (liquiritigenin) and 5-hydroxyflavanone, respectively (Shimada et al., 2003). The experiments in yeast or *E. coli* strains successfully demonstrate that they have significant differences in enzymatic activity (Shimada et al., 2003; Tian and Dixon, 2006).

Recently, microorganisms such as *Saccharomyces cerevisiae* and *Escherichia coli* have been adapted and engineered for heterologous isoflavonoid synthesis, overcoming the complexity associated with biosynthesis and accumulation in non-endogenous plants through advancements in synthetic biology. The *de novo* synthesis of parent isoflavonoids, such as genistein and quercetin, has been achieved in engineered yeast strains by overexpressing at least seven enzymes (PAL/TAL, 4CL, CHS, CHI, CHR, IFS, and IFD) (Trantas et al., 2009; Rodriguez et al., 2017). Co-cultivation of an IFS-expressing *S. cerevisiae* strain with a naringenin-producing *E. coli* strain resulted in the accumulation of genistein (6 mg/L) (Katsuyama et al., 2007). Additionally, *de novo* biosynthesis of bioactive isoflavonoids and the hops bioactive flavonoid xanthohumol has been achieved in yeast (Liu et al., 2021; Yang et al., 2024). These studies demonstrate that the optimal combination of key enzyme genes from various plants, the copy number of these genes, the physical distance between adjacent key enzymes, and the accommodation of membrane proteins by the endoplasmic reticulum are factors influencing the efficient synthesis of isoflavonoids. These are all important considerations for future *de novo* synthesis of (iso)flavonoidand other complex natural products.

## New technologies and resources

The CRISPR/Cas systems, known for their high efficiency and versatility, have found extensive applications in various plant genome editing and metabolic engineering endeavors (Wada et al., 2022). In *Fagopyrum tataricum*, the CRISPR/Cas9-mediated knockout of *FtMYB45* resulted in a reduction of flavonoids (Wen et al., 2022). In soybean, precise editing of key enzymes involved in isoflavonoid biosynthesis, including GmF3H1, GmF3H2, GmFNS-1, and Gm-IFS, was achieved through CRISPR/Cas9-directed mutagenesis (Zhang et al., 2020). This targeted mutagenesis led to a 2-fold increase in isoflavone content in soybean leaves and enhanced resistance to soybean mosaic virus. The study emphasized the roles of genes in isoflavonoid biosynthesis and phytohormones influencing growth effects (Mipeshwaree Devi et al., 2023).

Machine learning and multiomics approaches have also been incorporated into isoflavonoid research. Nearly thirty flavor molecule databases and various models have been identified (Kou et al., 2023). Over 1200 natural flavonoid compounds have been cataloged in the customized Flavonoid Astringency Prediction Database (FAPD, Guo et al., 2023). The establishment of this database facilitates an understanding of the relationship between the molecular structure of flavonoid compounds and their astringency in foods. Key genes in crops that influence astringency can be screened by integrating transcriptomic and metabolomic analyses. Subsequently, transgenic or gene editing approaches are utilized to verify the functions of these genes, facilitating the breeding of superior crop varieties that are both healthy and flavorful (Qin et al., 2022; Qiu et al., 2023). Given that the distribution of isoflavonoids and the genes involved in biosynthesis, regulation and transport are strongly induced by environmental factors, and exhibit tissue specificity and developmental stage specificity in leguminous plants. The integration of single-cell sequencing and spatial transcriptomics is poised to provide robust support for further elucidation and metabolic engineering of the isoflavonoid biosynthetic pathway. For instance, the iflavonoids in the roots of leguminous plants are closely related to the formation of root nodules. Through single-cell sequencing technology, researchers can analyze the gene expression patterns of specific cell types during the root nodule formation process at the single-cell level. Combined with transcriptomic sequencing, it is possible to further explore the gene expression patterns related to isoflavonoid synthesis, regulation, transport, and secretion.

## Conclusion and future prospects

Isoflavonoids play a crucial role in plant adaptation to complex environmental stimuli, with leguminous plant roots utilizing them to regulate nodule formation and influence overall growth. Consequently, exploring isoflavonoid metabolic engineering holds promise for genetic improvements in both legume and non-legume crops. However, it is important to note that changes in the composition and content of flavonoids, isoflavonoids, and lignin, which are interconnected through the phenylpropanoid pathway, could potentially have adverse effects on plants, such as reduced biomass, an imbalance between disease resistance and stress tolerance, and altered flavor. Therefore, it is necessary to consider the entire growth and developmental state of the plant, rather than focusing solely on changes in isoflavonoid content. Recent innovative approaches, exemplified by Sulis et al.’s work using a multiscale model of lignin biosynthesis, showcase effective multiplex CRISPR-editing strategies (Sulis et al., 2023). This enables the reduction of lignin levels in poplar without compromising growth, enhancing cell wall degradability, promoting cellulose utilization for papermaking and bioenergy production, and minimizing environmental impact. These advances provide valuable insights for precise and efficient crop genetic breeding.

What is the relationship between the molecular structure of isoflavonoid compounds and their bioactivity and flavor? Which genes determine the production of specific isoflavonoids? Which genes influence the transformation between free isoflavonoids and their modifications? What is the connection between the accumulation or secretion of plant isoflavonoids and the environment? Can the synthesis of isoflavonoids in non-leguminous plants achieve symbiosis with rhizobia to enhance nitrogen fixation? These are all subjects that require further research and exploration. Further exploration of diverse isoflavonoids and derivatives through metabolic engineering or synthetic biology requires the identification of key enzyme genes and regulators. Integration of machine learning and database predictions may expedite the discovery of additional enzymes and compounds. The future lies in the collaborative efforts of synthetic biology and metabolic engineering for efficient and sustainable isoflavonoid production. Advancements in multi-omics technologies are anticipated to unravel key insights into isoflavonoid biosynthesis, transport, and accumulation.

## Author contributions

LW: Funding acquisition, Resources, Writing – original draft, Data curation, Writing – review & editing. CL: Funding acquisition, Writing – original draft, Resources, Writing – review & editing. KL: Writing – original draft, Writing – review & editing.
